# Efficient Expression of Functionally Active Aflibercept with Designed N-glycans

**DOI:** 10.3390/antib13020029

**Published:** 2024-04-07

**Authors:** Tahereh Keshvari, Stanislav Melnik, Lin Sun, Ali Niazi, Farzaneh Aram, Ali Moghadam, Benjamin Kogelmann, Gordana Wozniak-Knopp, Somanath Kallolimath, Amin Ramezani, Herta Steinkellner

**Affiliations:** 1 Institute of Plant Biotechnology and Cell Biology, Department of Applied Genetics and Cell Biology, University of Natural Resources and Life Sciences BOKU Vienna, 1190 Vienna, Austria; tahereh.keshvari@yahoo.com (T.K.); lin.sun@boku.ac.at (L.S.); benjamin.kogelmann@boku.ac.at (B.K.); somanath.kallolimath@boku.ac.at (S.K.); 2 Institute of Biotechnology, Shiraz University, Shiraz 71441-65186, Iran; niazi@shirazu.ac.ir (A.N.); fa.aram@gmail.com (F.A.); ali.moghadam@shirazu.ac.ir (A.M.); 3 ACIB—Austrian Centre of Industrial Biotechnology, 1190 Vienna, Austria; 4 Institute of Molecular Biotechnology, Department of Biotechnology, University of Natural Resources and Life Sciences BOKU Vienna, 1190 Vienna, Austria; gordana.wozniak@boku.ac.at; 5 Shiraz Institute for Cancer Research, School of Medicine, Shiraz University of Medical Sciences, Shiraz 71348-14336, Iran

**Keywords:** aflibercept, plant expression, glycosylation, glycan engineering, Fc fusion, anti VEGF

## Abstract

Aflibercept is a therapeutic recombinant fusion protein comprising extracellular domains of human vascular endothelial growth factor receptors (VEGFRs) and IgG1-Fc. It is a highly glycosylated protein with five N-glycosylation sites that might impact it structurally and/or functionally. Aflibercept is produced in mammalian cells and exhibits large glycan heterogeneity, which hampers glycan-associated investigations. Here, we report the expression of aflibercept in a plant-based system with targeted N-glycosylation profiles. *Nicotiana benthamiana*-based glycoengineering resulted in the production of aflibercept variants carrying designed carbohydrates, namely, N-glycans with terminal GlcNAc and sialic acid residues, herein referred to as AFLI^GnGn^ and AFLI^Sia^, respectively. Both variants were transiently expressed in unusually high amounts (2 g/kg fresh leaf material) in leaves and properly assembled to dimers. Mass spectrometric site-specific glycosylation analyses of purified aflibercept showed the presence of two to four glycoforms in a consistent manner. We also demonstrate incomplete occupancy of some glycosites. Both AFLI^GnGn^ and AFLI^Sia^ displayed similar binding potency to VEGF_165_, with a tendency of lower binding to variants with increased sialylation. Collectively, we show the expression of functionally active aflibercept in significant amounts with controlled glycosylation. The results provide the basis for further studies in order to generate optimized products in the best-case scenario.

## 1. Introduction

Recombinant Fc-fusion proteins are receiving increased attention in the biopharmaceutical industry because of their respectable therapeutic efficiency [1], with aflibercept being a prominent example [2,3,4,5]. Aflibercept consists of extracellular domains of human VEGF receptors 1 and 2 fused with the Fc portion of human IgG1 (Figure S1) and acts as a soluble decoy receptor, interfering with the natural endogenous VEGF receptors [6,7]. This antibody-based drug is used in the treatment of various clinical conditions, such as retinal vascular diseases and certain cancers [8,9]. Biochemically, aflibercept is a heavily glycosylated dimeric protein, produced in CHO cells. An interesting feature of aflibercept is its ample glycosylation, with five conserved N-glycosylation sites (Figure S1). It was shown that the glycosylation pattern of recombinant aflibercept produced in mammalian cells is diverse, with up to 18 different N-glycan species [10]. Also, significant glycan heterogeneity between different batches was detected [11]. Both observations not only encumber its use due to the high standards required in therapeutic protein expression, but also hamper investigation of its glycan-associated features. Although it is expected that this important post-translational modification (PTM) has manifold impacts on protein structure and stability, pharmacokinetics, and/or interaction with VEGF, no explicit studies have been performed so far. Due to the complex glycosylation machinery of mammalian cells, this PTM is difficult to control or manipulate [12,13]. 

In recent years, engineered plants have been proven to efficiently express complex human proteins [14]. In particular, the development of transient expression tools that allow rapid protein production within days following the delivery of DNA constructs to plant leaves (mainly to those of *Nicotiana benthamiana* (*N. benthamiana*)) has significantly advanced the system [15]. In addition, the establishment of sophisticated tools that allow the expression of proteins with targeted human-type N-glycans at large homogeneity have contributed to the plant system receiving a great deal of attention [16]. More precisely, using genome editing and RNAi technology has resulted in the generation of plant lines (especially *N. benthamiana*) lacking plant-specific N-glycosylation and synthesizing simplified conserved N-glycans, namely, GnGn—the core structure preserved among higher eukaryotes [17,18,19]. These lines served as templates for further targeted N-glycan diversification, resulting in the reconstruction of the human sialylation pathway [20,21]. Such lines provide suitable platforms for the generation of complex human proteins with modified N-glycan structures and for subsequent evaluation of glycan-dependent structural and functional activities. While plant-based antibody glycan engineering has been repeatedly reported [16,22], such data are rare for engineered human proteins with a high degree of complexity, such as aflibercept.

The present study focuses on the expression of recombinant aflibercept with targeted and homogeneous N-glycosylation profiles. *N. benthamiana*-based glycoengineering [16] was used to generate two aflibercept glycoforms: one terminating with GlcNAc (GnGn) and one with sialic acid [18]. The glycan composition of protein A-purified recombinant aflibercept was elucidated by mass spectrometry (MS), its structural and functional integrity was determined by biochemical and immunological means.

## 2. Methods

### 2.1. Generation of Aflibercept Expression Vector

Aflibercept-coding DNA (1296 bp) was inserted into a magnICON^®^ vector pICH26211α—a derivative of pICH26211 (Icon Genetics GmbH (Halle/Saale, Germany)) [23] carrying a barley α-amylase signal sequence—using BsaI restriction sites, leading to the expression vector pICH26211α-aflibercept (Figure S2A). Sequence information is available in the Supplementary Materials Section (Figure S2B). The construct was transformed into *Agrobacterium fabrum* GV3101(pMP90) and the resulting strain used for the subsequent agroinfiltration experiments. 

### 2.2. In Planta Expression and Purification of Aflibercept

Glycoengineered *N. benthamiana* plants [18,24] were grown in a growth chamber under controlled conditions at 24 °C, 60% humidity, with a 16 h light/8 h dark photoperiod. 

For agroinfiltration, respective recombinant bacterial strains were grown at 29 °C for 24 h, centrifuged at 4000× *g* for 10 min at room temperature, and resuspended in infiltration buffer (10 mM MES, pH 5.6; 10 mM MgSO_4_). The optical density of each strain was measured by light absorption at 600 nm (OD_600_) of an adequate dilution. The final OD_600_ was set to 0.1 by dilution with infiltration buffer. The agrobacterial suspensions were delivered to leaves of 4–5-week-old plants using a syringe. The infiltrated leaves were harvested 4 days post-infiltration, flash-frozen in liquid nitrogen, and ground to fine powder. 

Total soluble protein (TSP) was extracted with extraction buffer (0.5 M NaCl, 0.1 M Tris, 1 mM EDTA, 40 mM ascorbic acid; pH 7.4) in a ratio of 1:2 *w*/*v* (fresh leaf weight: buffer) for 90 min at 4 °C on an orbital shaker. Subsequently, the solution was centrifuged twice at 14,000× *g* for 20 min at 4 °C and the supernatant was vacuum filtered using 8–12 µm and 2–3 µm filters (ROTILABO^®^ Typ 12A and 15A) (Karl Roth GmBH, Karlsruhe, Germany). 

To collect intercellular fluid, the infiltrated intact leaves were harvested 4 days post-infiltration (dpi) and submerged in a beaker containing IF extraction buffer (100 mM Tris·HCl, pH 7.5, 10 mM MgCl_2_, 2 mM EDTA). The leaves were infiltrated with the IF buffer by applying vacuum; excess liquid was removed with a paper towel and the leaves were placed inside separate 50 mL conical tubes equipped with a flat plastic mesh installed in the circular cross-section plane between the cylindrical and the conical parts of the tube to keep the leaves above the collected IF. The tubes were centrifuged at 800× *g* for 5 min at 4 °C to release the IF from the leaves.

To determine aflibercept expression, 10 µg of TSP extracted from the infiltrated leaf material was separated on a 12% SDS-PAA gel followed by immunoblotting using anti-human IgG (Goat anti-hIgG-HRPO, Thermofischer Scientific, Waltham, MA, USA Invitrogen 62-8420) at the dilution 1:5000. 

Recombinant proteins were purified by affinity chromatography using protein A (rProA Amicogen, Cat no: 1080025, Amicogen, Inc., Gyeongsangnam-do, Republic of Korea). The TSP extracts were loaded at a flow rate of 1.5 mL/min onto a manually packed column which was pre-equilibrated with 10 column volumes (CV) of PBS (137 mM NaCl, 3 mM KCl, 10 mM Na_2_HPO_4_, 1.8 mM KH_2_PO_4_; pH 7.4). Washing was carried out with 20 CV of PBS. Aflibercept was eluted in 1 mL fractions with 0.1 M Glycine·HCl (pH 3.0); eluates were immediately neutralized with 25 µL of 1 M Tris (pH 9.0) and dialyzed overnight against PBS.

The dialyzed and concentrated protein was subjected to size-exclusion chromatography using BioLogic DuoFlo FPLC system (Bio-Rad, South Granville, NSW, Australia) equipped with a QuadTec UV–vis detector (Bio-Rad) and Superdex 200 Increase 10/300 GL column (GE Healthcare Bio-Sciences AB, Uppsala, Sweden). Elution was performed at room temperature in an isocratic flow of PBS (137 mM NaCl, 2.7 mM KCl, 8.1 mM Na_2_HPO_4_, 1.76 mM KH_2_PO_4_, pH 7.4) at 0.75 mL/min and proteins were detected by UV absorbance at 280 nm.

### 2.3. N-Glycan Analyses

The N-glycosylation profiles of the purified aflibercept were determined by mass spectrometry (MS), as described previously [25,26]. Briefly, the 65 kDa band was excised from an SDS-PAGE gel, digested with trypsin, and analyzed with an LC-ESI-MS system (Thermo Orbitrap Exploris 480, Thermofisher Scientific, Waltham, MA, USA). The possible glycopeptides were identified as sets of peaks consisting of the peptide moiety and the attached N-glycan, varying in the number of HexNAc units, hexose, deoxyhexose, and pentose residues. Manual glycopeptide searches were performed using FreeStyle 1.8 (Thermofisher Scientific), deconvolution was performed using the extract function. The peak heights roughly reflect the molar ratios of the glycoforms. To estimate the unglycosylated fraction, the relative intensities of all glycoforms were summed up and subtracted from the total intensities. The nomenclature used was according to the Consortium for Functional Glycomics [27] and Proglycan [28]. For peptide mapping, the files were analyzed using PEAKS (Bioinformatics Solutions Inc., Waterloo, ON, Canada), which is suitable for performing MS/MS ion searches.

### 2.4. ELISA

An antigen binding assay was performed by following a previously published procedure (Sivertsen MS et al., 2018) with modifications. Human VEGF_165_ (a generous gift from Max Josef Kellner, IMBA, Vienna, Austria) was coated onto 96-well microplates (NUNC MaxiSorp™, Thermo Fisher Scientific, catalog No: M9410-1CS) with 50 µL/well at the concentration of 0.4 µg/mL (in PBS, pH 7.4) and incubated overnight. After four washes with PBS-T (PBS with 0.05% Tween 20), the plates were blocked with 4% fat-free milk powder dissolved in PBS-T for 1.5 h at RT. The aflibercept samples and Avastin (bevacizumab) were added (50 µL/well) as 2-fold serial dilutions (1000.0–1.0 ng/mL) in blocking solution and incubated at RT for 1.5 h. The plates were washed as described above; subsequently, 50 µL/well of human IgG-γ chain- HRPO antibody (Invitrogen 62-8420, 1:10,000 blocking solution) was applied and incubated for 1.5 h at RT. Detection was performed with 50 µL/well of 3,3′,5,5′-tetramethylbenzidine (Thermo Fisher, J61325. AU). The reaction was stopped with 50 µL/well of 2 M H_2_SO_4_ after 5–7 min of incubation. Light absorbance was measured at the 450 nm (reference 620 nm) wavelength using a Tecan Spark^®^ spectrophotometer (Tecan Group Ltd., Männedorf, Switzerland). All samples were analyzed at least in two technical replicates. EC_50_ values were calculated by non-linear regression of the blank-corrected data points based on a four-parametric log model with GraphPad Prism (version 9).

## 3. Results

### 3.1. Production of Recombinant Aflibercept in Glycoengineered N. benthamiana

**Figure 1:**
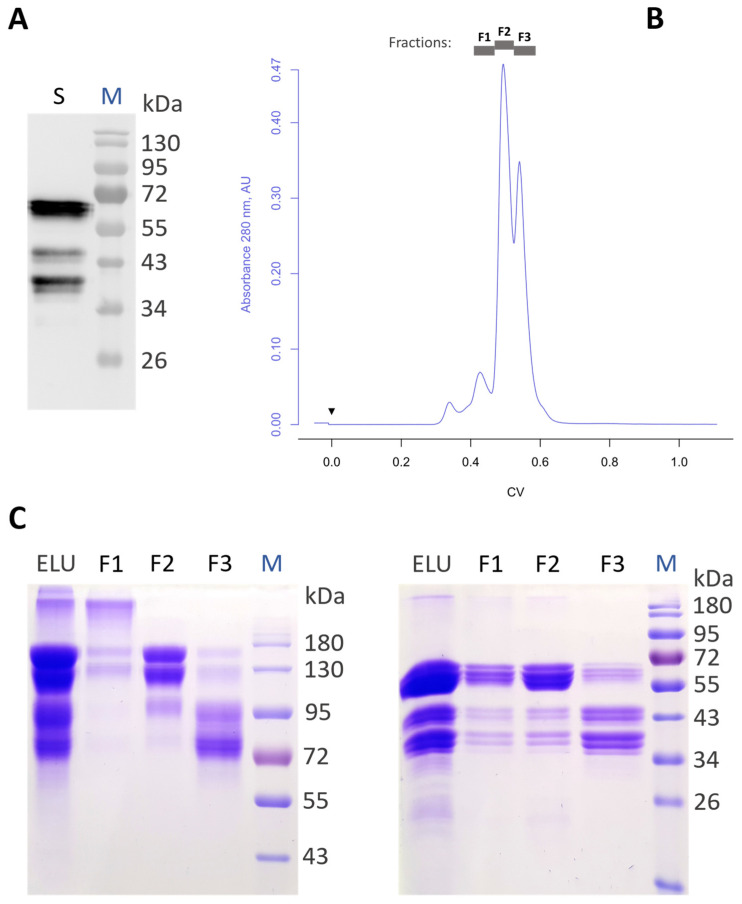
Aflibercept produced in *N. benthamiana*. (**A**) Western blot analysis of total soluble proteins (TSP) extracted from plants infiltrated with pICH26211α-aflibercept (lane S, ~10 µg of TSP was loaded). (**B**) SEC profile of protein A-purified aflibercept and location of collected fractions. (**C**) SDS-PAGE of protein A-purified aflibercept (ELU), and SEC fractions thereof (F1–F3), under non-reducing (**left**) and reducing (**right**) conditions; protein loading (ELU, F1, F2, F3): 6.5, 2, 4, 4 µg/lane.

A magnICON^®^ vector carrying an aflibercept DNA fragment (pICH26211α-aflibercept, Figure S2A) was delivered through agroinfiltration to leaf cells of a glycoengineered *N. benthamiana* that synthesizes complex N-glycans, lacking plant-specific xylose and fucose (GnGn structures) [18,24]. Aflibercept expression was monitored at 4 to 5 dpi in total soluble protein extracts. Immunoblotting under reducing conditions exhibited strong signals at ~65 kDa—the expected size of the full-length glycosylated monomer of aflibercept (assigned as AFLI^GnGn^), as well as additional signals in the range 35–45 kDa (Figure 1A). These fragments were allocated to degradation products and free Fc, as observed previously in experiments with plant-produced Fc fusions [29,30]. 

Determination of the expression level by semi-quantitative Western blotting (considering the 65 kDa product only; degradation products not included) showed that it exhibited an unusually high level of recombinant AFLI^GnGn^, amounting to 2 g/kg fresh leaf material (FLM; Figure S3). Subsequently, recombinant polypeptides were purified by protein A affinity followed by size-exclusion chromatography (SEC). The SEC chromatogram exhibited four major peaks, corresponding fractions of which were separately collected (F1–F3, Figure 1B). Further analysis of the collected fractions by SDS-PAGE revealed enrichment of the properly assembled full-length dimeric AFLI^GnGn^ in fraction 2 (F2) while F1 and F3 were ascribed to aggregates and degradation products, respectively (Figure 1C). Evidently, SEC enabled the separation of multimers and degradation products from AFLI^GnGn^ dimers, as confirmed by SDS-PAGE; the dimers accounted for approximately 50% of the protein A-purified products (F2 fraction). Polypeptides at position ~65 kDa (corresponding to AFLI^GnGn^) separated in three to four bands in close proximity and were analyzed by peptide mass fingerprinting. The integrity of AFLI^GnGn^ was confirmed (Figure S4), indicating that apparent molecular weight differences were associated with diverse N-glycosylation. The final yield of dimeric AFLI^GnGn^ (sample present in F2) was about 200 mg/kg FLM. Of note, while 2 g/kg FLM aflibercept expression levels were obtained for selected samples of single-infiltrated plants, only 0.4 g/kg of protein A-purified AFLI^GnGn^ was retrieved upon larger scale infiltrations involving whole leaf material from different plants. This corresponds to 20% of the yield achieved in single-leaf infiltration experiments. This massive loss is a consequence of the upscaling process, which includes the collection of a large number of leaves with different age and transformation extent, and the non-optimized subsequent downstream procedure. The inspection of the intercellular/apoplastic fluid (IF, representing secretome) of infiltrated leaves exhibited signals at a position around 65 kDa on SDS-PAGE (Figure S5), accompanied by bands at positions of 35–45 kDa, which correspond to degradation products, similar to what was observed for the variant isolated from TSP (Figure 1A,C—right). Intercellular fluid (equivalent to extracellular space) was used for subsequent protein A purification of aflibercept (IF-AFLI^GnGn^). In summary, aflibercept was efficiently expressed in plants and correctly assembled into dimers. 

### 3.2. Generation of Aflibercept with Different N-Glycosylation Profiles

An interesting feature of aflibercept is its high N-glycan content, with five conserved N-glycosites (GS, Figure S1). Overall, the N-glycans of aflibercept produced in mammalian cells account for about 15% of its molecular weight. Intensive investigations of the CHO-derived aflibercept have shown that it exhibits site-specific glycosylation, with large microheterogeneity [10]. GS1 to GS4 are efficiently sialylated, but differ in their sialylation content, while GS5 (which represents Fc GS) lacks this terminal sugar and instead carries mainly GlcNAc-terminated (G0F) structures. The majority of N-glycans are core-fucosylated. Glycoengineered plants were used in this study since they usually generate antibodies with largely homogeneous and reproducible GnGn structures, lacking plant-specific sugar residues [18,24,31]. Moreover, glycoengineered plant-derived IgGs often exhibit increased effector functions compared to orthologues produced in CHO cells or in wild-type plants [32,33].

**Figure 2:**
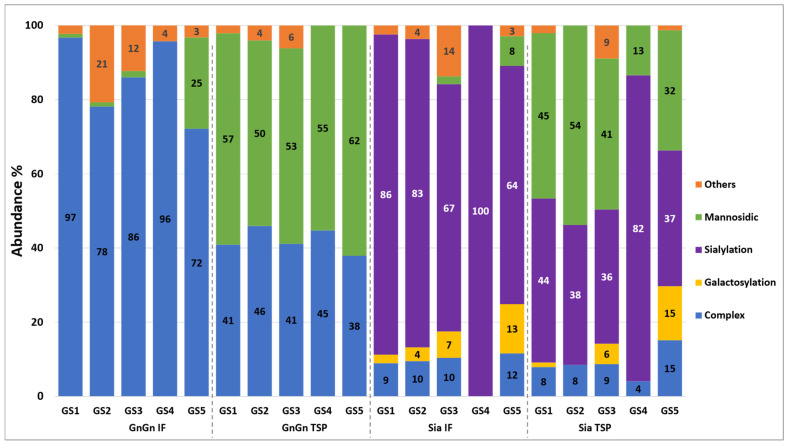
N-glycan composition of aflibercept expressed in *N. benthamiana* plants deficient in core xylose and fucose [18,24]. Four variants were analyzed by LC-ESI-MS: AFLI^GnGn^ and AFLI^Sia^ purified from TSP and IF. Histogram bars represent the relative abundance (%) of glycoforms present at each individual glycosite (GS1–GS5). For detailed information, see Table S1. Nomenclature according to Proglycan [28].

In order to determine the N-glycosylation status of plant-produced AFLI^GnGn^, liquid chromatography–electrospray ionization tandem mass spectrometry (LC-ESI-MS/MS) of the 65 kDa SDS-PAGE band was performed. To clearly characterize the glycan distribution, glycan profiles at each GS were identified and quantified at the glycopeptide level (Figure 2 and Table S1). All five GSs of protein A-purified AFLI^GnGn^ exhibited a mixture of mannosidic and GlcNAc-terminated complex N-glycans (predominantly GnGn). In addition, MS spectra from the IF-AFLI^GnGn^, representing the fully secreted fraction, were retrieved. Interestingly, only complex N-glycans (GnGn, GnM) were detected at all GSs (Figure 2 and Table S1). The GS occupancy studies revealed that GS1 and GS3 were fully occupied, while GS2, GS4, and GS5 exhibited underglycosylation, which was particularly pronounced at GS5 (~80%), corresponding to the Fc domain (Figure S6 and Table S1). 

Next, we envisaged the generation of aflibercept with sialylated N-glycans, since the mammalian-cell-produced therapeutic aflibercept (and presumably native VEGFR) are heavily sialylated [10]. For this, we co-delivered genes for the human sialylation pathway with aflibercept expression vectors to *N. benthamiana* plants, a procedure well established in the laboratory [20,34]. Infiltrated leaves were collected at 5 dpi, and aflibercept was subjected to protein A purification (assigned as AFLI^Sia^). MS spectra of the ~65 kDa SDS-PAGE band exhibited a significant portion of sialylated N-glycans (up to 82%), accompanied by mannosidic structures at all glycosites (Figure 2 and Table S1). In fact, the vast majority of GlcNAc-terminated N-glycans detected in AFLI^GnGn^ were further processed to sialylated forms (Figure 2 and Table S1). Notably, AFLI^Sia^ derived from IF carried mainly sialylated N-glycans, whereas mannosidic structures were not detected, in accordance with IF-AFLI^GnGn^. Collectively, we show the generation of glycoengineered aflibercept variants exhibiting largely homogeneous glycosylation profiles. 

### 3.3. VEGF Binding Activity of Aflibercept Glycoforms

**Figure 3:**
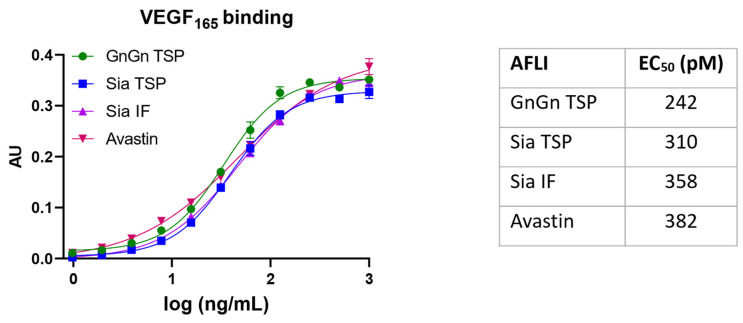
Binding ELISA of aflibercept variants to VEGF_165_. GnGn TSP: AFLI^GnGn^ isolated from TSP; Sia TSP and Sia IF: AFLI^Sia^ isolated from TSP and IF, respectively.

The mechanism of action of aflibercept is to inhibit the natural VEGF receptor by binding to secretory VEGF, thereby efficiently reducing/blocking angiogenesis induced by this glycoprotein cytokine. Aflibercept binds VEGF in a unique manner, by not only blocking the amino acids necessary for binding to VEGFR1/R2, but also occluding the heparin-binding site on VEGF_165_ [35,36]. Thus, the functional integrity of plant-produced aflibercept was determined by an ELISA-based binding assay using recombinant VEGF_165_. To determine a possible impact of glycosylation, three variants were tested: AFLI^GnGn^, TSP-AFLI^Sia^, and IF-AFLI^Sia^ (Figure 3 and Figure S7). The retrieved EC_50_ values are similar (242, 310, and 358 pM, respectively) and demonstrate efficient binding. However, there is a tendency of reduced binding depending on the sialylation content. Of note, the binding values are similar to bevacizumab (Avastin, 382 pM)—a highly active therapeutic mAb that also targets VEGF. Binding activity remains unchanged after 6 months storage at −20 °C (Figure S8).

## 4. Discussion

This study demonstrates the rapid expression of functionally active aflibercept with targeted glycosylation profiles (AFLI^GnGn^ and AFLI^Sia^). While mAbs and Fc-fusion proteins have been successfully produced in plants previously [37], the expression levels were usually lower than we observed for recombinant aflibercept, with around 2 g per kg FLM in small-scale experiments. To our knowledge, this is amongst the highest expression level reported for a complex human protein in plants [38,39], which makes plant-based aflibercept production highly interesting for the biopharmaceutical industry. Notably, the expression might be increased even further by the use of *N. benthamiana*-optimized expression constructs, and the addition of gene silencing inhibitors, like p19. A possible downside of extreme expression levels could be underglycosylation of target proteins, as observed for high IgG production using the magnICON^®^ system [40]. Indeed, a surprising observation in this study was the large variation in GS occupancies. While GS1 and GS3 were efficiently occupied, GS2, GS4, and GS5 were incompletely glycosylated—with the effect being most pronounced at GS5 (up to 80% non-glycosylated fraction), representing the Fc GS. This has not been observed in aflibercept produced in mammalian cells, where, except for GS2 (20–25% underglycosylation), all other GSs are fully occupied [10]. Underglycosylation might impact on the structure of the protein, with so-far-unknown consequences. While it is well documented that low Fc glycosylation impairs antibody effector functions (like ADCC and CDC), it is important to note that the activity mechanism of aflibercept does not require such activities. Efficacies rather rely on blocking/binding to VEGF [2]. If and how different GS occupancies affect in vivo activities need to be determined. In case higher glycosylation occupancies turn out to be advantageous, glycosylation efficiency might be increased by the co-expression of oligosaccharyltransferase (OST) domains, responsible for the transfer of the oligosaccharide (Glc3Man9GlcNAc2) to nascent polypeptides. Overexpression of OST increases GC occupancies of recombinantly expressed polypeptides significantly [41,42]. 

Another peculiarity of plant-produced aflibercept is the relatively high proportion of mannosidic structures in variants purified from TSP. Such incompletely processed glycans are not present in the IF-derived versions, which exhibit only virtually fully processed GnGn and sialylated N-glycans. High amounts of mannosidic structures (>50%) were reported on other Fc fusions expressed in plants [43] and might be the consequence of insufficient secretion. Such structures were detected on CHO-produced aflibercept, albeit to a much lesser extent [11,44]. Our results show that mannosidic structures of aflibercept do not impact on VEGF binding; however, some recombinant fusion proteins containing high mannose-type glycans exhibit reduced serum half-life [45]. Notably, plant-produced IgG antibodies carrying exclusively oligomannosidic structures exhibited a decreased murine half-life compared to the same antibody carrying complex N-glycans [46].

Importantly, here we demonstrate the reliable production of aflibercept with a well-defined glycosylation pattern, i.e., GlcNAc- or sialic acid-terminated N-glycans. In particular, the IF-derived fractions provide large glycan homogeneity, carrying one to two dominant glycoforms: either GnGn/GnM or sialylated N-glycans (NaNa/NaM). This opens a possibility for more detailed investigation on the impact of this dominant PTM in future studies. Our results are in stark contrast to mammalian-cell-produced counterparts that show significant glycan-associated microheterogeneity due to the large endogenous glycosylation repertoire of mammalian cells [10,11]. In addition, the presence of N-glycolylneuraminic acid (NeuGc) glycans, which may mediate adverse immune reactions in antibody therapeutics [11], have also been detected on the sites of the VEGFR1 and VEGFR2 domains. Such glycan residues are not synthesized in plants.

Biochemical studies of plant-produced aflibercept did not reveal obvious differences among the individual glycoforms. AFLI^GnGn^ and AFLI^Sia^ exhibited similar expression levels and assembly behaviors. In accordance, no clear differences in VEGF_165_ binding were observed between sialylated and nonsialyltaed aflibercept variants. Nevertheless, a slight tendency in VEGF_165_ binding could be extracted, ranging from 242 to 358 pM, depending on the sialylation content. These results are consistent with studies showing that VEGFR2 glycans capped with sialic acid oppose ligand-mediated VEGFR2 activation, whereas the uncapped asialo-glycans favor activation of this receptor [47]. Whether glycan-dependent binding can also be observed with more complex VEGFs, such as that present in vivo, remains to be determined. Another advantage of highly sialylated aflibercept may be associated with PK values, as efficient terminal sialylation can extend the serum half-life of glycoproteins [45]. 

A challenge in this study was the modest purification yield of dimeric aflibercept, which accounted for approx. 0.2 g/kg FLM. Nonetheless, this number exceeds by far other plant-produced Fc-fusion products that require SEC polishing [48]. However, a more comprehensive comparison on purification yields is difficult, as most related studies do not provide appropriate numbers. The reduction in product yield upon process upscaling is a well-known phenomenon, observed not only in plants [38,49] but in other expression systems as well. Still, the low yield of purification (10% of the expression levels achievable in individual leaf samples) indicates the pressing need for optimization of upscaling and purification procedures of this highly interesting pharmaceutical product. This is particularly important since analyses by Data Bridge Market Research suggest that the aflibercept market is expected to undergo a CAGR of 5.4% during a forecast period of 2030 [50]. It should be noted that the current mammalian cell expression is an expensive process and the resulting high production costs of aflibercept significantly contribute to the enormous price of the final product [51,52]. 

In summary, we were able to produce aflibercept with highly controlled glycosylation profiles, exhibiting targeted, largely homogeneous, N-glycosylation. Currently, it is totally unknown how different glycosylation affects efficacy of aflibercept. The present work provides the basis for further more advanced functional studies, like in vivo performance evaluation. The plant-based system allows the generation of well-defined forms that go beyond the present study [40]. Aflibercept produced in mammalian cells exhibits a collection of different glycoforms, some of them being more active than others. This might generate a non-optimized product, with possible unwanted side effects, particularly in high-dose applications. Understanding and controlling the glycosylation of aflibercept is crucial for ensuring its efficacy and safety in the treatment of various medical conditions. In this context, plant-based expression, which allows tight control over glycosylation [16,37], might contribute to the production of variants with optimized efficiencies, thereby reducing adverse reactions and financial burden. In addition, the sustainable and cost-effective plant expression [53] might contribute to the more equal distribution of high-tech products among low- and middle-income countries.

## Data Availability

Data are contained within the article and Supplementary Materials.

## Supplementary Materials

The following supporting information can be downloaded at: https://www.mdpi.com/article/10.3390/antib13020029/s1. Figure S1: Schematic structure of aflibercept homodimer. One of the two polypeptide chains is highlighted in color. VEGFR1-D2: second domain of human VEGF receptor 1; VEGFR2-D3: third domain of human VEGF receptor 2; GS1-GS5: glycosites 1 to 5; Figure S2: Construction of plant overexpression vector for transient expression of aflibercept. A. Cloning procedure. Act2_P: A. thaliana actin 2 gene promoter; TVCV genetic elements are depicted in light grey (Ω: omega leader; RdRP: RNA-dependent RNA polymerase; MP: movement protein; 3’NTR: 3’ non-translated region), bent arrows indicate subgenomic promoters; SP: barley α-amylase signal peptide; VEGFR1-D2: second domain of human VEGF receptor 1; VEGFR2-D3: third domain of human VEGF receptor 2; H-CH2-CH3*: hinge region, human IgG1 constant heavy chain domains 2 and 3, stop codon (*), respectively; nos_T: nopaline synthase terminator. RB, LB: right and left T-DNA borders, respectively; bacterial genes for plasmid replication and maintenance are shown in dark grey (ColE1 ori, RK2 oriV: origins of replication, trfA: replication initiation protein for RK2 OriV; aphA: aminoglycoside O-phosphotransferase APH(3’)-IIIa conferring resistance to kanamycin). B. Aflibercept CDS amino acid sequence details; N-glycosite sequons are highlighted in bold; Figure S3: Semi-quantitative Western Blot to determine expression level of aflibercept. Left: different amount of total soluble protein (TSP) was loaded; right: quantified IgG1 standard served as control. M: protein marker. * and ** correspond to 55 and 70 kDa, respectively; Figure S4: Peptide mass-fingerprint analysis and sequence coverage of recombinant aflibercept. Sequence covered is highlighted in gray boxes in bold and the observed individual peptides are represented as blue lines underlying their respective matching sequence; carbamidomethylated cysteines and oxidized methionines are highlighted in red and orange, respectively; Figure S5: SDS-PAGE under reducing conditions of IF sample (A) isolated from leaves of *N. benthamiana* expressing aflibercept; (B) aflibercept (AFLI^GnGn^) from IF after protein A purification; S: sample, M: protein molecular weight marker; staining: Coomassie Brilliant Blue R-250; Figure S6: Comparison of glycosite occupancies of different AFLI variants; Figure S7: SDS-PAGE of protein A- and SEC-purified aflibercept variants used for ELISA. M: molecular weight marker; GnGn_TSP: AFLI^GnGn^ isolated from TSP; Sia_TSP: AFLI^Sia^ isolated from TSP; Sia_IF: AFLI^Sia^ isolated from IF; staining: Coomassie Brilliant Blue R-250; Figure S8: Stability assay of plant produced AFLI. Binding ELISA of aflibercept (AFLI^GnGn^) to VEGF165 after 6 months storage at −20 °C. Positive control: avastin; negative control: irrelevant IgG1; Table S1: Relative N-glycan distribution of different AFLI glycovariants on the 5 glycosylation sites.
