# Supplementary material for: Efficient Expression of Functionally Active Aflibercept with Designed N-glycans
Source: Antibodies (Basel). 2024 Apr 7;13(2):29. doi: 10.3390/antib13020029 (PMC11036266; doi:10.3390/antib13020029)
Supplement: Supplementary file 1 [file antibodies-13-00029-s001.zip › antibodies-2799356-supplementary.pdf]

Supplementary Materials

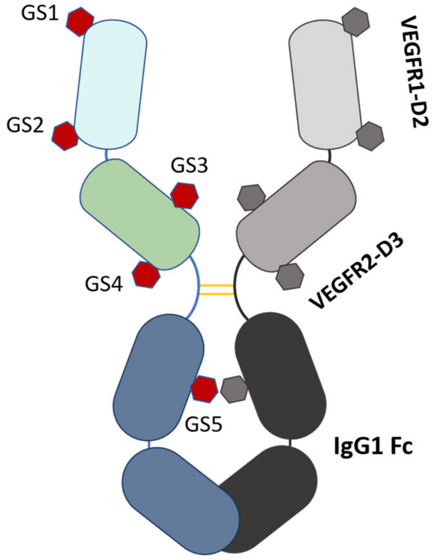

Glycosite	Asn	Glyco-Peptide
GS1	N36	VTSP<u>N</u>ITVTLK
GS2	N68	GFIIS<u>N</u>ATYK
GS3	N123	LVL<u>N</u>CTAR
GS4	N196	<u>N</u>STFVR
GS5	N282	EEQY<u>N</u>STYR

Figure S1. Schematic structure of aflibercept homodimer. One of the two polypeptide chains is highlighted in color. VEGFR1-D2: second domain of human VEGF receptor 1; VEGFR2-D3: third domain of human VEGF receptor 2; GS1-GS5: glycosites 1 to 5.

A

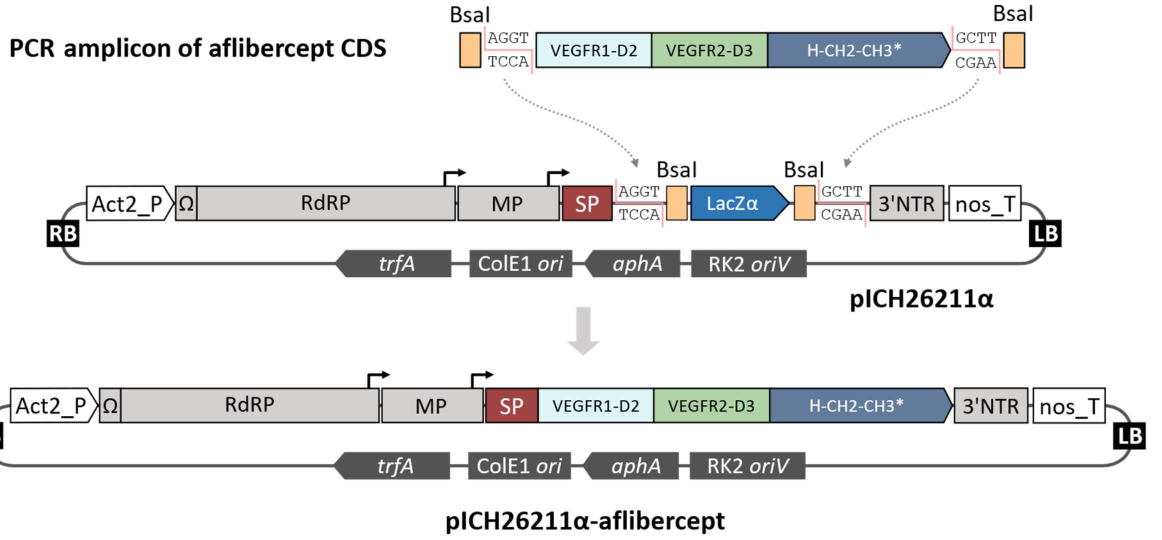

B

SDTGRPFVEMYSEIPEIIHMTEGRELVIPCRVTSP **NIT**VTLKKFPLDTLIPDGKRIIWDSRKGFIIS**NAT**YKEIGLL
TCEATVNGHLYKTNYLTHRQTNTIIDV VLSPSHGIELSVGEKLVL**NCT**ARTELNVGIDFNWEYPSSKHQHKKL
VNRDLKTQSGSEMKKFLSTLTIDGVTRSDQGLYTCAASSGLMTKK **NST**FVRVHEKDKTHTCPPCPAPELLGG
PSVFLFPPKPKDTLMISRTPEVTCVVVDVSHEDPEVKFNWYVDGVEVHNAKTKPREEQY **NST**YRVVSVLTV
LHQDWLNGKEYKCKVSNKALPAPIEKTISKAKGQPREPQVYTLPPSRDELTKNQVSLTCLVKGFYPSDIAVE
WESNGQPENNYKTTPPVLDSDGSFFLYSKLTVDKSRWQQGNVFSCSVMHEALHNHYTQKSLSLSPGK *

- VEGFR1-D2·VEGFR2-D3·hinge·IgG1Fc

Figure S2. Construction of plant overexpression vector for transient expression of aflibercept. A. Cloning procedure. Act2_P: *A. thaliana* actin 2 gene promoter; TVCV genetic elements are depicted in light grey (Ω: omega leader; RdRP: RNA-dependent RNA polymerase; MP: movement protein; 3'NTR: 3' non-translated region), bent arrows indicate subgenomic promoters; SP: barley α-amylase signal peptide; VEGFR1-D2: second domain of human VEGF receptor 1; VEGFR2-D3: third domain of human VEGF receptor 2; H-CH2-CH3*: hinge region, human IgG1 constant heavy chain domains 2 and 3, stop codon (*), respectively; nos_T: nopaline synthase terminator. RB, LB: right and left T-DNA borders, respectively; bacterial genes for plasmid replication and maintenance are shown in dark grey (ColE1 ori, RK2 oriV: origins of replication, trfA: replication initiation protein for RK2 OriV; aphA: aminoglycoside O-phosphotransferase APH (3')-IIIa conferring resistance to kanamycin). B. Aflibercept CDS amino acid sequence details; N-glycosite sequons are highlighted in bold.

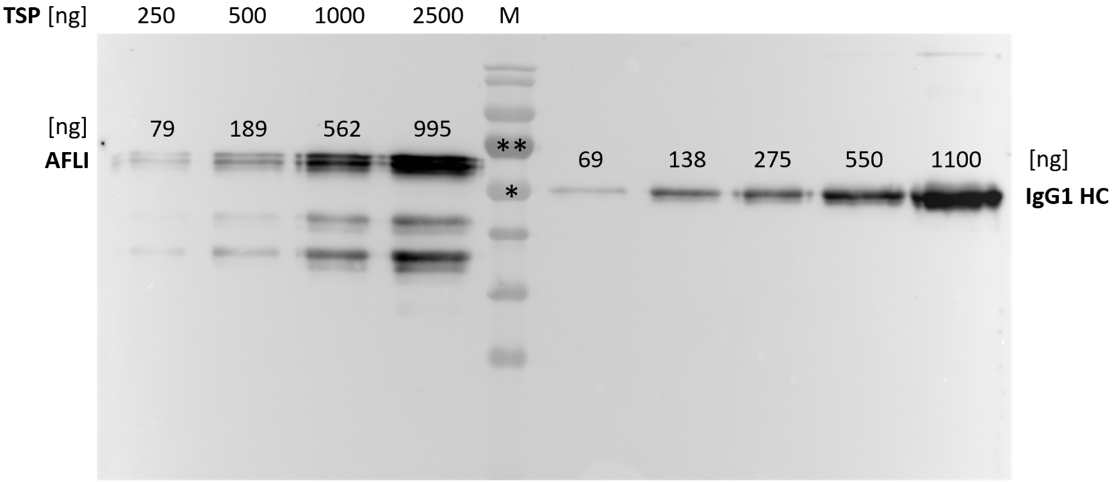

Figure S3. Semi-quantitative Western Blot to determine expression level of aflibercept. Left: different amount of total soluble protein (TSP) was loaded; right: quantified IgG1 standard served as control. M: protein marker. * and ** correspond to 55 and 70 kDa, respectively.

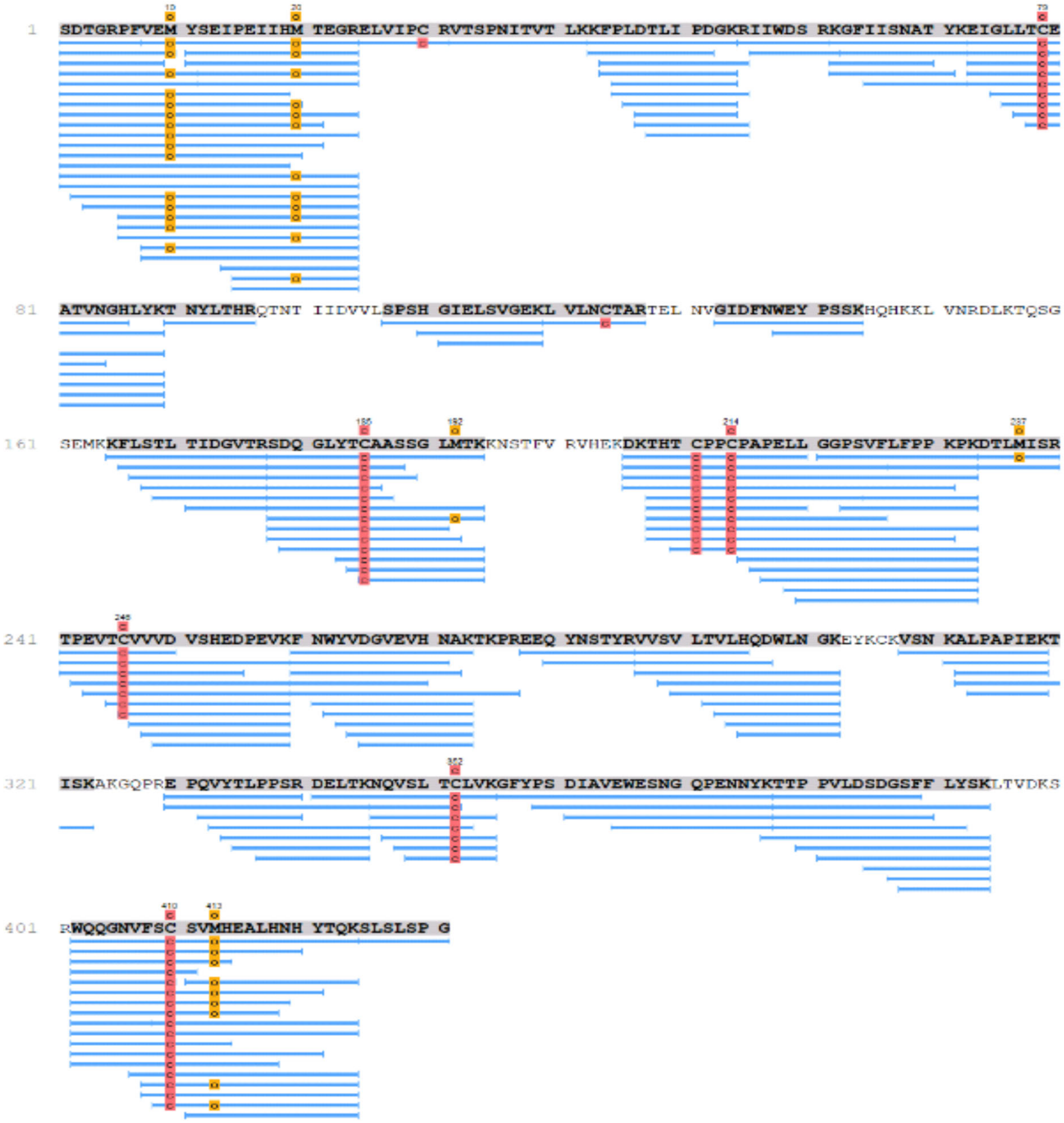

Figure S4. Peptide mass-fingerprint analysis and sequence coverage of recombinant aflibercept. Sequence covered is highlighted in gray boxes in bold and the observed individual peptides are represented as blue lines underlying their respective matching sequence; carbamidomethylated cysteines and oxidized methionines are highlighted in red and orange, respectively.

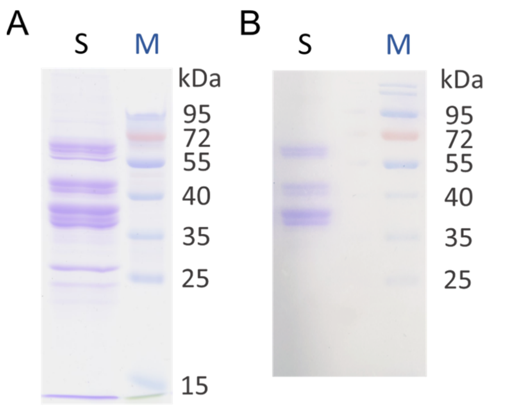

Figure S5. SDS-PAGE under reducing conditions of IF sample (A) isolated from leaves of *N. benthamiana* expressing aflibercept; (B) aflibercept ($AFLI^{GnGn}$) from IF after protein A purification; S: sample, M: protein molecular weight marker; staining: Coomassie Brilliant Blue R-250.

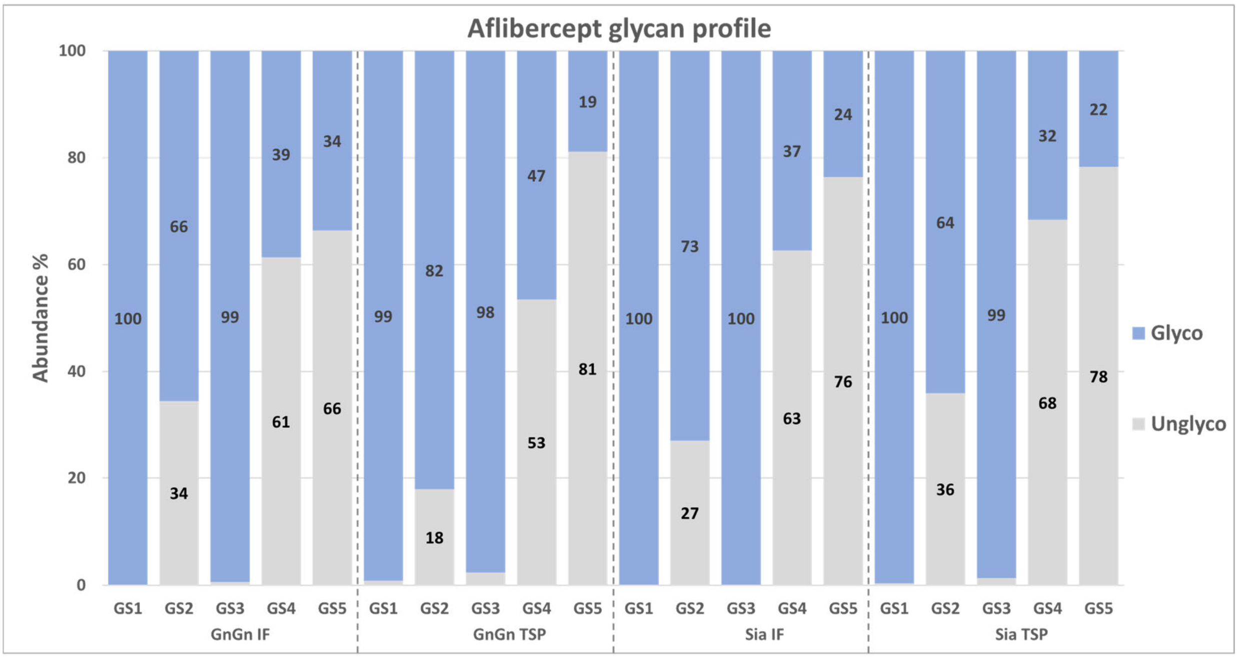

Figure S6. Comparison of glycosite occupancies of different AFLI variants.

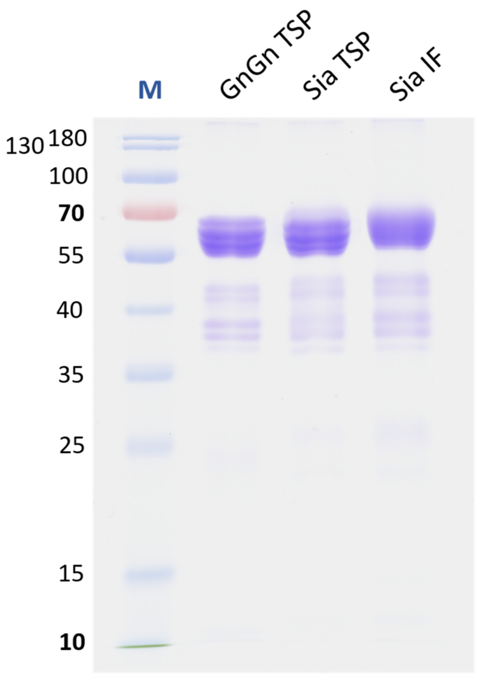

Figure S7. SDS-PAGE of protein A- and SEC-purified aflibercept variants used for ELISA. M: molecular weight marker; GnGn_TSP: AFLI^{GnGn} isolated from TSP; Sia_TSP: AFLI^{Sia} isolated from TSP; Sia_IF: AFLI^{Sia} isolated from IF; staining: Coomassie Brilliant Blue R-250.

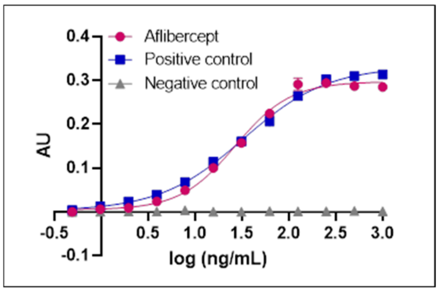

Figure S8: Stability assay of plant produced AFLI. Binding ELISA of aflibercept (AFLI^{GnGn}) to VEGF165 after 6 months storage at -20oC. Positive control: avastin; negative control: irrelevant IgG1.

Table S1. Relative N-glycan distribution of different AFLI glycovariants on the 5 glycosylation sites.

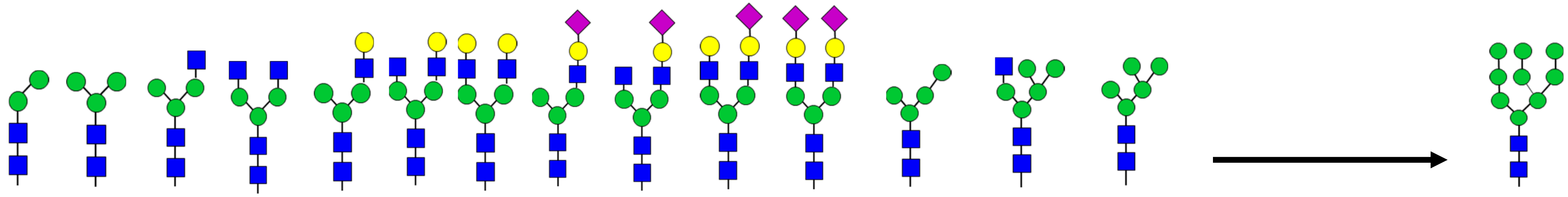

	GS	MU	MM	GnM	GnGn	AM	AGn	AA	NaM	NaGn	NaA	NaNa	Man4	Man5Gn	Man5	Man6	Man7	Man8	Man9
GnGn IF	1	0.0	2.2	15.4	81.3	0.0	0.0	0.0	0.0	0.0	0.0	0.0	0.0	0.1	0.0	0.0	0.8	0.3	0.0
	2	14.9	5.2	11.7	66.5	0.0	0.0	0.0	0.0	0.0	0.0	0.0	0.0	0.0	0.0	0.0	0.1	0.7	1.0
	3	0.0	11.7	61.5	24.5	0.0	0.0	0.0	0.0	0.0	0.0	0.0	0.4	0.6	0.3	0.0	0.3	0.3	0.4
	4	0.0	4.3	9.9	85.9	0.0	0.0	0.0	0.0	0.0	0.0	0.0	0.0	0.0	0.0	0.0	0.0	0.0	0.0
	5	0.0	0.0	7.0	65.2	0.0	0.0	0.0	0.0	0.0	0.0	0.0	1.0	3.2	5.8	4.2	7.9	4.9	0.8
GnGn TSP	1	0.0	2.1	7.2	33.7	0.0	0.0	0.0	0.0	0.0	0.0	0.0	2.2	0.0	4.6	3.9	23.6	16.8	6.0
	2	0.0	4.1	8.0	38.0	0.0	0.0	0.0	0.0	0.0	0.0	0.0	1.6	0.0	3.5	3.3	6.7	0.7	34.1
	3	0.0	6.2	28.6	12.5	0.0	0.0	0.0	0.0	0.0	0.0	0.0	0.9	0.0	5.3	4.0	4.3	13.4	24.8
	4	0.0	0.0	4.4	40.3	0.0	0.0	0.0	0.0	0.0	0.0	0.0	0.0	0.0	2.6	2.3	2.8	16.3	31.2
	5	0.0	0.0	3.9	34.0	0.0	0.0	0.0	0.0	0.0	0.0	0.0	1.4	0.0	3.9	4.1	7.7	11.8	33.1
Sia IF	1	0.0	2.5	2.6	6.3	2.3	0.0	0.0	16.6	3.8	4.6	61.3	0.0	0.0	0.0	0.0	0.0	0.0	0.0
	2	0.0	3.7	2.9	6.6	3.7	0.0	0.0	32.1	5.0	0.0	46.0	0.0	0.0	0.0	0.0	0.0	0.0	0.0
	3	2.9	10.4	8.7	1.7	7.1	0.0	0.0	61.6	0.5	0.0	4.6	0.5	0.5	0.5	0.3	0.3	0.0	0.6
	4	0.0	0.0	0.0	0.0	0.0	0.0	0.0	20.6	1.3	3.0	75.0	0.0	0.0	0.0	0.0	0.0	0.0	0.0
	5	0.0	2.8	2.9	8.7	3.7	6.1	3.5	52.7	10.9	0.0	0.6	0.0	0.0	0.0	1.9	3.7	2.5	0.0
Sia TSP	1	0.4	2.1	2.1	5.7	1.3	0.0	0.0	7.5	1.5	2.9	32.0	2.1	0.0	3.1	3.2	15.3	14.0	6.8
	2	0.0	0.0	2.2	6.3	0.0	0.0	0.0	12.0	1.7	3.6	20.5	0.0	0.0	2.8	4.9	4.2	8.6	33.3
	3	0.0	7.2	8.4	1.8	4.9	0.3	0.4	32.8	0.0	0.7	2.5	1.7	0.4	2.9	3.6	3.9	0.0	28.7
	4	0.0	0.0	0.0	4.1	0.0	0.0	0.0	22.4	0.0	3.6	56.4	0.0	0.0	0.0	0.0	3.2	0.0	10.2
	5	0.0	1.3	2.6	12.5	2.2	7.7	4.7	19.4	4.1	12.3	0.9	0.0	0.0	0.0	3.6	5.6	5.2	18.0
